# The Identification of a Strain for the Biological Purification of Soy Molasses to Produce Functional Soy Oligosaccharides and Optimize Purification Conditions

**DOI:** 10.3390/foods13020296

**Published:** 2024-01-17

**Authors:** Ran Yang, Jinghao Ma, Zechen Wang, Yihua Du, Shubin Tian, Guangsen Fan, Xiaoyan Liu, Chao Teng

**Affiliations:** 1 School of Food and Health, Beijing Technology and Business University, Beijing 100048, China; yangran@btbu.edu.cn (R.Y.); 15032049018@163.com (J.M.); norn139@163.com (Z.W.); 15810226593@163.com (Y.D.); liuxiaoyan8112@163.com (X.L.); tengchao@th.btbu.edu.cn (C.T.); 2 Sweet Code Nutrition and Health Institute, Zibo 256306, China; sweetcodelab@163.com; 3 Key Laboratory of Green Manufacturing and Biosynthesis of Food Bioactive Substances, China General Chamber of Commerce, Beijing Technology and Business University, Beijing 100048, China; 4 Beijing Engineering and Technology Research Center of Food Additives, Beijing Technology and Business University, Beijing 100048, China

**Keywords:** soy molasses, stachyose, raffinose, biological purification, *Wickerhamomyces anomalus*

## Abstract

Soy molasses is rich in oligosaccharides like sucrose, stachyose, and raffinose, with stachyose and raffinose being functional oligosaccharides. Harnessing soy molasses for the production of functional soy oligosaccharides (FSO) can significantly elevate its value. Biological purification, a method leveraging the selective utilization of different carbon sources by microorganisms, allows for the specific removal of sucrose from soy molasses while preserving stachyose and raffinose, thereby increasing the FSO content. This research identified a yeast named YT312 with strong purification capabilities for soy molasses and optimized the purification conditions. The study revealed that yeast YT312 was *Wickerhamomyces anomalus*, exhibiting a broad range of growth temperatures and pH levels alongside a high tolerance to glucose, sucrose, and NaCl. Through single-factor and orthogonal experiments, it was established that under specific conditions—0.375% inoculum size, 30 °C fermentation temperature, 150 rpm shaking speed, 10-fold dilution ratio, pH of 7, and 12 h of fermentation—sucrose was completely removed from soy molasses, while functional raffinose and stachyose were retained at rates of 96.1% and 90.2%, respectively. Consequently, *W. anomalus* YT312 displayed exceptional characteristics for the biological purification of soy molasses and the production of FSO.

## 1. Introduction

Soy molasses is a byproduct and yellow-brown viscous substance produced during the production of soy protein concentrate (SPC) using the alcohol method [1,2]. Statistics show that for every three to four tons of SPC produced, around one ton of soy molasses is generated. This means that 100,000 tons of soy molasses are produced every year globally [3,4]. Studies revealed that soy molasses comprises a variety of nutrients and useful compounds, giving it a complex composition. The main components were carbohydrates, including oligosaccharides and monosaccharides, which account for 58–65% of the dry weight. Additionally, soy molasses contained approximately 6–8% protein, 5–8% lipids, 7–9% minerals, 6–15% soyasaponins, 1.5–2.5% soy isoflavones, and other organic compounds such as 4–6% phenolic acids [2,4,5,6,7]. However, soy molasses was previously considered trash and discarded or utilized as cheap animal feed, which led to a large loss of resources or the contamination of the environment despite the abundance of these vital components [8]. In recent years, with the increasing awareness of environmental protection and the demand for sustainable development strategies, countries have intensified efforts to reuse soy molasses. It was utilized as a biomass resource for the extraction and isolation of functional components or as a nutrient source for microbial growth and biotransformation. For example, a combination of solvents and ultrasound treatment has been used to extract soy isoflavones with high antioxidant activity from soy molasses [9,10,11]. Resin adsorption and elution methods, as well as ultrasound-assisted techniques, were utilized for the extraction and purification of soyasaponins [12,13]. Phospholipids have been produced by using an organic solvent extraction process after acid precipitation [14]. Additionally, kynurenic acid, enzymes, bacterial cellulose, bio-methane, ethanol, and cells have been produced by microbial fermentation using soy molasses as a source of carbon and nitrogen [15,16,17,18,19]. The extensive and valuable use of soy molasses was greatly impacted by these investigations.

Functional soy oligosaccharides (FSO) are a group of soluble oligosaccharides found in soybeans that are not easily utilized by the human body. The main components of FSO are raffinose and stachyose, which make up approximately 1% and 4% of soy, respectively [20]. In the past, these oligosaccharides were considered the primary cause of gastrointestinal bloating, leading to efforts in soybean processing to eliminate or cultivate soybean varieties without these oligosaccharides [20,21,22,23]. However, as research into the functions of different oligosaccharides progressed, scientists discovered that although the human body could not directly utilize FSO, such as raffinose and stachyose, they were utilized by beneficial bacteria in the intestine, such as *Bifidobacterium* and *Lactobacillus*, promoting their growth and producing short-chain fatty acids that provide various health benefits. These benefits included lowering the pH in the intestines, regulating blood lipid and blood sugar levels, enhancing immune function, and preventing diseases and aging [24,25,26,27]. Currently, FSO are widely used as a novel kind of prebiotic in the production of beverages, ice cream, and bread, as well as in pharmaceuticals, cosmetics, and animal feed [28].

The direct isolation and purification of soy oligosaccharides from soybeans was relatively costly. However, they were extracted and purified from byproducts of soy processing, preserving the original value of soybeans while transforming waste into valuable resources, thereby increasing the value of the soy industry chain. Among the various byproducts of soy processing, soy molasses was particularly suitable as a raw material to produce FSO. Soy molasses, which was produced during the processing of SPC, has a higher concentration of FSO compared to soybean raw materials, with raffinose and stachyose contents ranging from 4 to 25.5% and 15.5 to 34.2%, respectively. This made soy molasses more suitable for the extraction and purification of FSO than soybean raw materials themselves [2]. However, there were certain difficulties in directly extracting raffinose, stachyose, and other FSO from soy molasses. This was because soy molasses often contained high levels of sucrose (usually 18.5–32%), requiring multiple steps of chromatographic purification and other fine purification methods to obtain high-purity FSO. This increased production costs and limited their application [2,5].

A crucial concept for differentiating and classifying microbial species according to their capacity to metabolize various carbon sources is that different microorganisms often have distinct carbon source metabolism profiles [29,30,31,32]. With this knowledge, different microorganisms can be used for the pre-treatment of soy molasses to break down sucrose and utilize it through the microorganisms’ specific carbon source metabolism while preserving FSO such as raffinose and stachyose to the greatest extent possible. This pre-treatment method greatly reduces the difficulty of extracting and separating FSO and improves their production efficiency. In fact, researchers explored this method and achieved good results [33,34,35,36]. For example, Zhao et al. [33] used *Saccharomyces cerevisiae* C to biologically ferment soy molasses, removing all sucrose from soy molasses under optimal conditions, with a retention rate of FSO of 60.9%. Cui et al. [34] used a yeast strain to ferment soy molasses, with a residual sucrose content of only 8.76%, while the content of raffinose and stachyose was almost unchanged, with retention rates of 99.61% and 95.72%, respectively. Liu et al. [35] purified soy molasses using *S. cerevisiae* 1607, and the results showed a sucrose removal rate of up to 90%, while the retention rates of raffinose and stachyose were 85.7% and 94.3%, respectively. Liu et al. [36] also achieved similar results using *S. cerevisiae* 103, with a residual sucrose content of 10.3%, and the retention rates of raffinose and stachyose were 81.0% and 92.5%, respectively. In general, these studies yielded positive outcomes in the purification of soy molasses and the biological production of FSO. However, the strains used in these studies were limited, especially *S. cerevisiae*. It was worth exploring whether other microbial strains also have good potential for purifying soy molasses and producing FSO. In our prior research, we isolated and maintained over 200 strains of microorganisms from traditional fermented foods for the biological purification of soy molasses to produce FSO [37]. Among them, strain YT312, obtained from a high-temperature Daqu, showed outstanding biological purification effects, and some of its physiological characteristics were different from *S. cerevisiae*. As a result, the strain was determined, and the fermentation conditions were adjusted in this article to enable the biological purification of soy molasses to yield FSO.

## 2. Materials and Methods

### 2.1. Chemicals and Materials

Soy molasses was provided by Shandong Rongzheng Chemical Co., Ltd. (Linyi, China). Sucrose, raffinose, stachyose, and manninotriose standards were purchased from Sigma-Aldrich Co., Ltd. (Shanghai, China). Fungal genomic DNA extraction kit was purchased from Beijing Solarbio Technology Co., Ltd. (Beijing, China). Other reagents used were domestically produced biological or analytical reagents. Strain YT312 was obtained by our research team from the environment of Baijiu brewing. Yeast extract peptone dextrose (YPD) medium, Wallerstein laboratory nutrient (WL) medium, and other identification media for strains were prepared according to the methods described by Fan et al., Kurtzman et al., Buchana et al., and Dong et al. [38,39,40,41]. The fermentation medium was processed according to our previous method, as follows: soy molasses was diluted with distilled water 8-fold, and the pH was adjusted to 6.0; then, it was divided into 30 mL per bottle (250 mL) and sterilized at 110 °C for 10 min [37].

### 2.2. Identification and Biochemical Characteristics of Strain YT312

#### 2.2.1. Morphological Observation

Strain YT312, stored in glycerol tubes, was streaked onto YPD and WL agar plates. The plates were then incubated at 30 °C for 72 h. After incubation, the colony morphology was observed. Then, a small number of cells from a single colony of strain YT312 was gently and evenly spread in a sterile water droplet on a glass slide. After fixation, crystal violet and methylene blue staining solution was used for staining, and the cells were observed under a high-power microscope (objective lens × eyepiece: 100 × 50).

#### 2.2.2. Physiological and Biochemical Characteristics

A total of 50 μL of the strain YT312 was stored in a glycerol tube, inoculated into YPD liquid medium, and activated at 30 °C and 180 rpm for 18 h. After activation (the cell amount was 2.6 × 10^7^ cells/mL by using a hemocytometer), physiological and biochemical characteristics were determined using methods such as sugar fermentation test, carbon source assimilation test, nitrogen source assimilation test, hydrogen sulfide production test, indole test, methyl red test, Voges–Proskauer test, citrate test, starch hydrolysis test, urea test, gelatin hydrolysis test and litmus milk test, as described by Buchana et al. and Dong et al. [40,41].

#### 2.2.3. Molecular Identification

Once the strain YT312 was activated, as previously mentioned, 50 μL of the suspension was added to the YPD medium and cultured for 24 h at 30 °C and 180 rpm. The genomic DNA of strain YT312 was extracted according to the instructions of the fungal DNA kit. The extracted total DNA was used as a template, and 26S rDNA universal primers (NL1: 5′-GCATATCAATAAGCGGAGGAAAAG-3′ and NL4: 5′-GGTCCGTGTTTCAAGACGG-3′) (synthesized by Sangon Biotech Co., Ltd., Shanghai, China) were used to amplify the 26S rDNA D1/D2 region sequence [42]. The PCR reaction system included 2.5 μL of LA PCR Buffer (Takara, Tokyo, Japan), 1 μL of each primer, 2 μL of dNTP (Takara, Tokyo, Japan), 0.2 μL of LAtaq enzyme (Takara, Tokyo, Japan), and 2 μL of DNA, and ddH_2_O was added to make up a total of 25 μL. The PCR amplification program was as follows: initial denaturation at 94 °C for 5 min, denaturation at 94 °C for 30 s, annealing at 58 °C for 30 s, extension at 72 °C for 1 min for a total of 30 cycles, followed by a final extension at 72 °C for 10 min. The PCR amplification products of strain DNA were subjected to electrophoresis using a 1% agarose gel for analysis. The amplified PCR product with the correct molecular weight was sent to Sangon Biotech Co., Ltd. (Shanghai, China) for sequencing. The sequence profile software BioEdit 7.0.9 (Borland Software Corporation, Scotts Valley, CA, USA) was used to proofread the sequence, referring to the forward sequence profile. The corrected 26S rDNA D1/D2 sequence was compared with known yeast sequences in the GenBank nucleic acid sequence database by homologous sequence alignment to determine the similarity between the test strain and known yeast sequences.

#### 2.2.4. Strain Performance Determination

After being activated as described in Section 2.2.2, the performance of YT312, including growth temperature range (20 °C, 25 °C, 30 °C, 35 °C, 40 °C, 45 °C, and 50 °C), growth pH range (1–12), sucrose tolerance (23%, 28.6%, 33.3%, 37.5%, 41.2%, 44.4%, and 47.3% *w*/*w*), glucose tolerance (23%, 28.6%, 33.3%, 37.5%, 41.2%, 44.4%, and 47.3% *w*/*w*), and NaCl tolerance (0%, 5%, 10%, 15%, 20%, 25%, and 30% *w*/*v*), was measured as reported previously [39].

#### 2.2.5. Determination of Strain Growth Curve

The growth curve of strain YT312 was determined using two methods: static and shaking (180 rpm). The activated strain YT312 was inoculated into YPD liquid medium at 0.1% inoculum size. The suspension was incubated in a static incubator at 30 °C and on a shaking incubator at 180 rpm. Samples were taken at 0 h, 3 h, 6 h, 9 h, 12 h, 15 h, 18 h, 21 h, 24 h, 27 h, 30 h, 33 h, and 36 h to measure the strain density.

### 2.3. Optimization of Fermentation Conditions by Single-Factor Experiments

The yeast cell suspension was precultured, and then 1% of the suspension was added to the fermentation medium as an inoculant. The blend was then incubated for 12 h at 200 rpm at 30 °C. The fermentation liquid was centrifuged at 10,000× *g* for 10 min, and the recovered supernatant was then filtered through a 0.22 μm filter membrane and put into a liquid chromatography vial for high-performance liquid chromatography (HPLC) analysis, as mentioned in Section 2.6. Based on the conditions mentioned above, a single-factor experiment (Table 1) was conducted step by step to investigate the effects of fermentation conditions (inoculum size, temperature, shaking speed, dilution ratio, pH, and time) on the purification of soy molasses by strain YT312.

### 2.4. Optimization of Fermentation Conditions by Orthogonal Experiment

Based on the single-factor experiments, the relative optimal levels of inoculum size (A), temperature (B), shaking speed (C), dilution ratio (D), pH (E), and time (F) were used to design an orthogonal experiment. Each factor was set at 3 levels (1, 2, and 3), and an L18 (3)^7^ orthogonal experimental design was selected. The combinations of orthogonal experimental groups are shown in Table 2.

### 2.5. Verification of Fermentation Conditions for Soy Molasses Purification

Based on the optimal combination levels obtained from the orthogonal experimental results, verification experiments were conducted under the optimal fermentation conditions to determine the retention rate of sucrose, stachyose, raffinose, and the content of manninotriose for the biological purification of soy molasses.

### 2.6. Determination of Sucrose, Stachyose, Raffinose, and Manninotriose

The content of sucrose, stachyose, raffinose, and manninotriose in the fermented soy molasses was determined using HPLC [43]. The determination conditions were as follows: the chromatographic column used was a Cosmosil-packed column (Sugar-D 4.6ID × 250 mm, Nacalai Tesque, Inc., Kyoto, Japan), the column temperature was set to 30 °C, the mobile phase was a 70% acetonitrile solution, the detector used was a Shimadzu refractive index detector (RID-10A) (Shimadzu Corporation, Kyoto, Japan), and the flow rate was set to 0.5 mL/min. Sucrose standard solutions with concentrations of 1 mg/mL, 2 mg/mL, 3 mg/mL, 4 mg/mL, and 5 mg/mL were prepared, and the peak areas of each concentration of sucrose standard solution were determined using HPLC to construct a sucrose standard curve. The same method was used to construct standard curves for stachyose, raffinose, and manninotriose. The content of the four sugar components in the samples was calculated based on the respective standard curves.

### 2.7. Determination of Cell Density

Strain biomass was calculated using the turbidity method (measuring absorbance at 560 nm wavelength). The amount of yeast cells was counted using a hemocytometer.

### 2.8. Determination of Comprehensive Index

As this study involved multiple response values, for the convenience of statistical calculation and selection of the optimal fermentation conditions, a weighted comprehensive scoring method was used to analyze the experimental results. Firstly, the sucrose retention rate, stachyose retention rate, raffinose retention rate, and manninotriose content in the experimental results were determined and calculated. Subsequently, the range was calculated using the maximum and minimum values of the experimental results and then transformed into dimensionless numbers through “range normalization” to assess the quality of the experimental results. Finally, a weighted comprehensive score was assigned to the “range normalization” to transform the multi-index problem into a single-index problem for analysis, thereby simplifying the index to determine the optimal conditions.

#### 2.8.1. Determination of Range Normalization Values

In this study, the sucrose retention rate was a negative indicator, where a smaller value was better. The raffinose retention rate and the stachyose retention rate were positive indicators in this study, where a larger value was better. Although manninotriose was also a functional soy oligosaccharide, it was derived from the conversion of stachyose in soy molasses by microbial fermentation. As mentioned earlier, since the stachyose retention rate was considered a positive indicator, the content of manninotriose was treated as a negative indicator. A smaller value indicated a higher stachyose retention rate in the comprehensive index. For the sucrose retention rate, raffinose retention rate, stachyose retention rate, or content of manninotriose, the indicator value obtained in the t-th experiment group (where t was the experiment group number) was denoted as Zt (t = 1, 2, 3, …, n). Here, the minimum value was denoted as Z(min), and the maximum value was denoted as Z(max). The range R1 was calculated as shown in Equation (1).

R1 = Z(max) − Z(min)(1)

The “range normalization” value ω or σ for each measurement value Zt of the negative indicator sucrose retention rate or manninotriose content, respectively, was defined as shown in Equation (2).

ω or σ = (Z(max) − Zt)/R1 = (Z(max) − Zt)/(Z(max) − Z(min))(2)

The “range normalization” value τ or φ for each measurement value Zt of the positive indicator raffinose retention rate or stachyose retention rate, respectively, was defined as shown in Equation (3).

τ or φ = (Zt − Z(min))/R1 = (Zt − Z(min))/(Z(max) − Z(min))(3)

#### 2.8.2. Determination of Weights

The determination of weights corresponding to each indicator generally relies on experience or the importance of experimental indicators. Based on practical experience and theoretical analysis, the proportions of various sugars in soy molasses were considered to ensure a total weight of 1. Combining the experimental purpose, actual situation, and experimental experience, the weights for each sugar corresponding to the experimental indicators were assigned.

We define the weight of sucrose as 0.5 and the total weight of raffinose and stachyose as 0.5, taking into consideration that our objective to produce FOS from the biological purification of soy molasses is to remove as much sucrose as possible while optimizing the retention of raffinose and stachyose. Next, we determine the weights of raffinose and stachyose, respectively, by using Equation (4) based on the proportionate connection between their concentrations in soy molasses. Specifying the weight of manninotriose as 0.01, which is derived from stachyose and has a content of 0 in soy molasses, allows for determining the actual weight of stachyose by subtracting 0.01 from the value obtained from Equation (4).

αi = Ci/(C1 + C2)(4)

where C1 was the concentration of raffinose in soy molasses; C2 was the concentration of stachyose in soy molasses; i = 1 or 2; α1 was the weight of raffinose.

Then, the weight of stachyose can be determined based on Equation (5).

β = α2 − 0.01(5)

#### 2.8.3. Determination of Comprehensive Index

The weighted “range normalization” values K for each indicator were computed in accordance with Equation (6), which represents the comprehensive index of this experiment, based on the “range normalization” values and weights established using the aforementioned approach.

K = 0.5ω + α1τ + βφ + 0.01σ(6)

Through the above analysis, the determination of the optimal combination of multiple indicators and the ranking of experimental factors was transformed into the measurement of the weighted comprehensive value K (single indicator) to determine the optimal combination of experimental factors and the ranking of experimental factors.

### 2.9. Data Processing

Each experiment was conducted in triplicate, and the experimental data were processed using Excel 2016 (Microsoft, Redmond, WA, USA), SPSS 18.0 (IBM Corp., New York, NY, USA), and OriginPro 9.1 (OriginLab, Northampton, MA, USA).

## 3. Results and Discussion

### 3.1. Identification and Biological Characteristics of Strain YT312

#### 3.1.1. Identification of Strain YT312

1. Observation of colony morphology and cell structure

**Figure 1:**
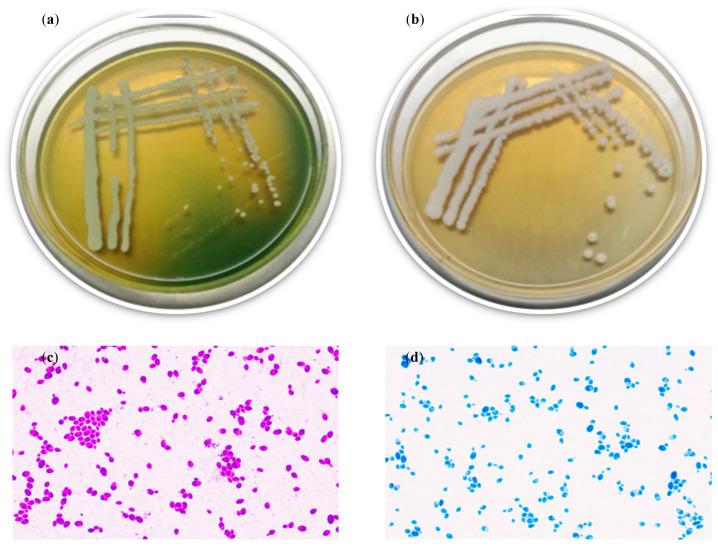
Colony morphology of strain YT312 on WL agar medium (**a**) and YPD agar medium (**b**), and its cell morphology (stained with crystal violet (**c**) and methylene blue (**d**); objective lens × eyepiece 100 × 50).

Figure 1a depicts the growth of strain YT312 on the WL agar medium. The colonies were flat, white with a slight gray-green color, small, and the color of the medium changed from dark green to light yellow where the strain grew. On the YPD agar medium, the colonies appeared milky white, large, and flat (Figure 1b). Examining the strain under a microscope, we found that its cells were ovoid or ellipsoidal, budding at one end, and without hyphae. (Figure 1c,d), which was similar to the reported colony morphology of yeast YF1503 by Fu et al. [44]. Therefore, it could be concluded that the strain YT312 exhibited typical yeast colony and cell morphologies.

2. Physiological and biochemical characteristics

Based on the sugar fermentation test (Table 3), yeast YT312 produced organic acids and gas when fermenting sucrose as a carbon source. However, when fermenting maltose, galactose, and glucose as carbon sources, it produced only organic acids without gas production. When fermenting xylose, lactose, arabinose, and sorbose as carbon sources, yeast YT312 produced neither organic acids nor gas. Assimilation tests for different carbon sources indicated that yeast YT312 could utilize sucrose, maltose, xylose, lactose, galactose, arabinose, sorbose, glucose, soluble starch, mannitol, citric acid, trehalose, ethanol, ribose, glycerol, or fructose as the sole carbon source for growth but could not utilize formic acid as the sole carbon source. Nitrogen assimilation tests showed that yeast YT312 could utilize various nitrogen sources, including urea, potassium nitrite, ammonium sulfate, potassium nitrate, and L-phenylalanine, as the sole nitrogen source for growth. In the hydrogen sulfide test, no blackening of the medium was observed, indicating that yeast YT312 did not have the ability to produce hydrogen sulfide by decomposing sulfur-containing amino acids in the medium. In the indole test, the medium turned red, indicating that yeast YT312 had the ability to decompose tryptophan in the medium and produce indole. In the methyl red test, the medium turned red, indicating that yeast YT312 metabolized glucose to produce a large number of acidic products, consistent with the results of the sugar fermentation test. In the Voges–Proskauer test, the medium did not turn red, indicating that although yeast YT312 converted glucose into pyruvic acid during metabolism, it did not have the ability to further metabolize pyruvic acid to produce acetyl methyl alcohol. In the citrate test, the medium turned blue, indicating that strain YT312 decomposed citrate into carbonate, making the medium alkaline, consistent with the results of the assimilation test for carbon sources. In the starch hydrolysis test, no transparent zone was observed in the medium. Included in the results of the assimilation test for carbon sources, it was indicated that the amylase activity produced by this yeast was low, and its ability to hydrolyze starch was weak. In the urea test, it showed a negative reaction, but in the nitrogen assimilation test, it could grow when urea was the sole nitrogen source. This indicated that although yeast YT312 could utilize urea, it could not produce urease to decompose urea to ammonia gas. The gelatin hydrolysis test also showed a negative reaction, indicating that this yeast did not have the ability to hydrolyze gelatin. In the litmus milk test, the medium turned red, indicating that yeast YT312 fermented lactose and produced acid, which was inconsistent with the results of the sugar fermentation test. This discrepancy might be due to differences in the composition of the two media or the variability in lactose fermentation. The physiological and biochemical characteristics of yeast YT312 were similar to those of *Wickerhamomyces anomalus* [45].

3. Molecular identification

After yeast YT312’s genomic DNA was extracted, the D1/D2 sequence of the 26S rDNA was amplified by using certain primers. The DNA sequence obtained from sequencing (Accession: OR673958) was then compared to the NCBI database using Blast (comparison conducted on 16 October 2023). The results revealed the highest identification with *W. anomalus* AUN-F44 (Accession: MK744056.1), *W. anomalus* KELXL-3 (Accession: JX049429.1), and *W. anomalus* S1B2-9 (Accession: MG773340.1), with a similarity of over 99%. Based on comprehensive morphological observations, physiological and biochemical characteristics, and molecular biology identification, yeast YT312 was identified as *W. anomalus*.

#### 3.1.2. Biological Characteristics of *W. anomalus* YT312

**Figure 2:**
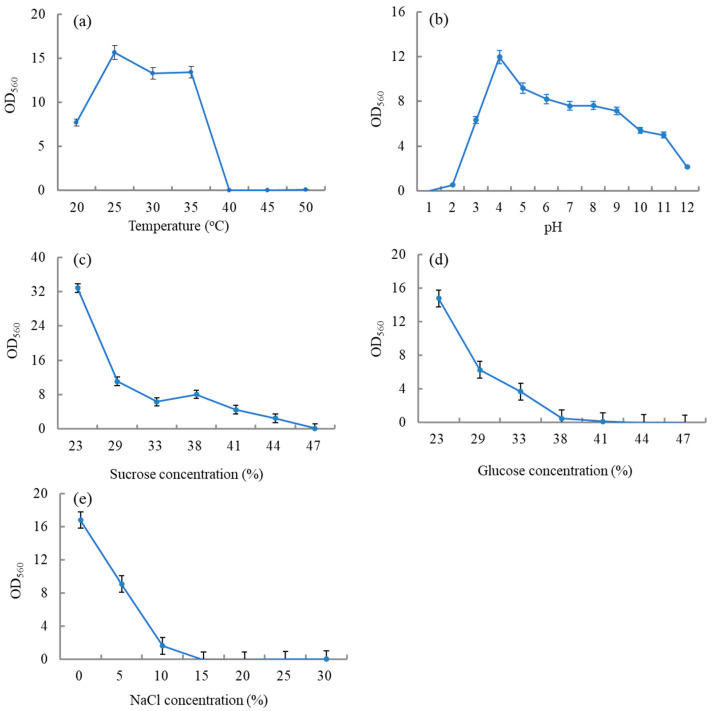
Biological characteristics of *W. anomalus* YT312. (**a**) growth temperature, (**b**) pH, (**c**) sucrose tolerance, (**d**) glucose tolerance, and (**e**) sodium chloride tolerance.

As shown in Figure 2a, the optimal growth temperature for *W. anomalus* YT312 was 25 °C, with a maximum tolerance temperature between 35 and 40 °C, which was similar to the growth characteristics of most yeast [39]. *W. anomalus* YT312 exhibited a wide pH growth range, capable of growing within a pH range of 2–12 (Figure 2b). Furthermore, it demonstrated strong acid tolerance, similar to *Pichia kudriavzevii* YF1702 and *Clavispora lusitaniae* YX3307 [29,46]. This might be attributed to its adaptation to the long-term survival environment. The optimal pH for the growth of *W. anomalus* YT312 was 4.0, slightly lower than that of most yeast [29,39]. As depicted in Figure 2c,d, the growth of *W. anomalus* YT312 was increasingly inhibited with the increase in glucose and sucrose concentrations. It exhibited a maximum tolerance to a glucose concentration of 41.2% and sucrose concentration of 47.3%. The glucose tolerance was slightly lower compared to *P. kudriavzevii* YF1702 and *C. lusitaniae* YX3307 [29,46]. With increasing NaCl concentration, the growth of *W. anomalus* YT312 was also increasingly inhibited, with a maximum tolerance to NaCl concentrations reaching 10%. This was similar to *P. kudriavzevii* YF1702 but slightly lower than *W. anomalus* Y3604 and *C. lusitaniae* YX3307 [29,39,46].

#### 3.1.3. Growth Curve of *W. anomalus* YT312

**Figure 3:**
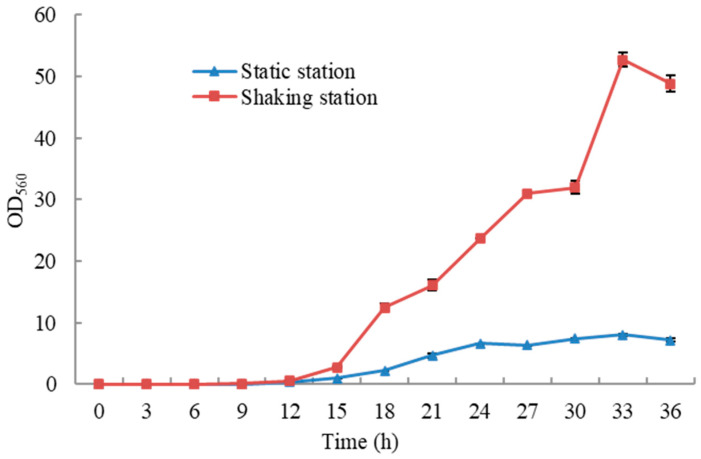
Growth curves of *W. anomalus* YT312 under shaking and static incubation conditions.

Figure 3 shows the growth curves of *W. anomalus* YT312 under two different incubation conditions: shaking and static. It was observed that under both shaking and static incubation conditions, with an inoculum size of 0.1%, *W. anomalus* YT312 had a lag phase of 12 h, followed by entering the logarithmic growth phase. However, the logarithmic phase was longer when incubated under shaking conditions, lasting 33 h, while it was shorter under static incubation, ending at 24 h. This difference was mainly due to the difference in the oxygen supply capacity between the two incubation methods [47]. Continuing the incubation under shaking conditions, *W. anomalus* YT312 did not exhibit a distinct stationary phase or have a shorter stationary phase. It entered the death phase within the sampling time intervals. On the other hand, under static cultivation, it showed a clear stability period, lasting up to 33 h before entering the death phase. This was primarily due to the difference in oxygen supply between the two incubation methods, leading to differences in the utilization rate of nutrients in the medium by *W. anomalus* YT312. In the shaking incubation, nutrients were rapidly and completely utilized, resulting in a rapid depletion of nutrients and quick entrance into the death phase. In contrast, under static incubation, the limited oxygen supply restricted growth, but nutrients were abundant, resulting in a more pronounced stability period until nutrients were gradually depleted, leading to the onset of the death phase. Additionally, it was also observed that the cell density was significantly higher under shaking incubation than under static incubation. This was attributed to the differences in metabolic pathways and the accumulation of different metabolic byproducts under the two incubation conditions, which had varying impacts on yeast cell proliferation.

### 3.2. Optimization of Fermentation Conditions for Soy Molasses Purification by Single-Factor Experiments

#### 3.2.1. Effect of Inoculum Size of *W. anomalus* YT312 on Soy Molasses Purification

**Figure 4:**
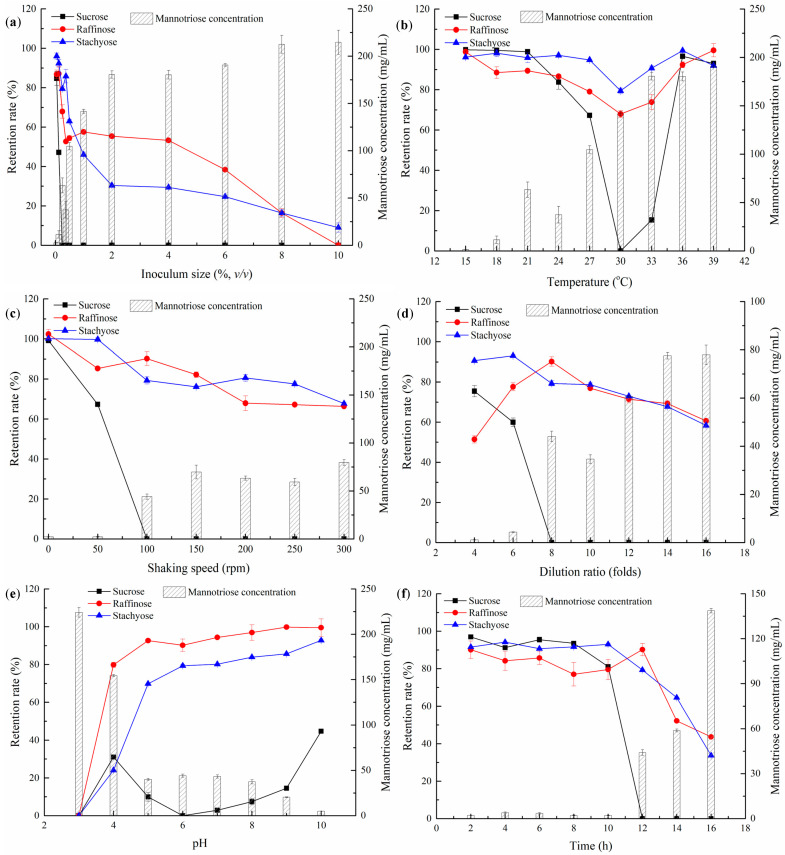
Effect of fermentation conditions of *W. anomalus* YT312 on the composition of fermented soy molasses. (**a**) Inoculum size, (**b**) temperature, (**c**) shaking speed, (**d**) dilution ratio, (**e**) pH, and (**f**) time.

Inoculum size affects the growth cycle of microorganisms [46]. Under the fixed fermentation time, different inoculum sizes led to different growth stages of microorganisms, resulting in different metabolic activities. In this study, different inoculum sizes caused variations in the sugar composition of the *W. anomalus* YT312 metabolic fermentation system. Under an appropriate inoculum size, *W. anomalus* YT312 primarily utilized sucrose in the fermentation system while minimizing or rarely utilizing raffinose and stachyose. Figure 4a illustrates how the sucrose content of the soy molasses decreased steadily as the inoculum size increased. When the inoculum size was 0.25%, there was almost no sucrose present in soy molasses. Raffinose and stachyose also showed a decreasing trend, especially when the inoculum size exceeded 0.25%; both exhibited a significant decrease, while there was a corresponding increase in manninotriose. This was because sucrose was the preferred sugar source for *W. anomalus* YT312. When sucrose was abundant, it was preferentially utilized by *W. anomalus* YT312 to provide energy and a carbon source for its growth and reproduction. This was confirmed by the retention rates of raffinose and stachyose, as well as the manninotriose content at inoculum sizes of 0.05% and 0.125%. When the inoculum size was smaller, the sucrose content was sufficient to provide the necessary energy and act as a carbon source for *W. anomalus* YT312 growth, resulting in less utilization of raffinose and stachyose. However, as the inoculum size increased, yeast growth and reproduction accelerated, and the sucrose concentration became insufficient to provide adequate energy and act as a carbon source for *W. anomalus* YT312. In order to sustain its development and reproduction at this stage, the yeast is required to use raffinose and stachyose in soy molasses. As a result, the retention rates of raffinose and stachyose decreased significantly, and a large amount of manninotriose was produced as stachyose was degraded and utilized. By analyzing the influence of inoculum size on the four sugar contents in soy molasses, it was concluded that an inoculum size of 0.25% (*v*/*v*) effectively removed sucrose from soy molasses and ensured a high retention rate of the functional oligosaccharides raffinose and stachyose at 67.9% and 79.4%, respectively. Therefore, an inoculum size of 0.25% was suitable for the *W. anomalus* YT312 bio-purification of soy molasses. This was lower than the optimal inoculum sizes for other yeast strains, such as *S. cerevisiae* 1607, *S. cerevisiae* 103, and maltose yeast, mainly due to the different adaptability of yeast strains to their environment [35,36,48].

#### 3.2.2. Effect of Fermentation Temperature on Soy Molasses Purification by *W. anomalus* YT312

Microbial growth and reproduction have their suitable temperature range. Under appropriate temperatures, microbial growth and reproduction are rapid, which is conducive to exerting their corresponding functions [49]. As shown in Figure 4b, at lower temperatures (15–18 °C), *W. anomalus* YT312 grew slowly, and the retention rates of sucrose, raffinose, and stachyose in soy molasses were higher. As the temperature increased, the metabolic activity of *W. anomalus* YT312 enhanced, and the consumption rate of sugar in soy molasses increased, resulting in a decrease in the content of sucrose, raffinose, and stachyose in soy molasses. When the fermentation temperature was 30 °C, *W. anomalus* YT312 consumed almost all the sucrose in soy molasses. And there were still high retention rates of stachyose and raffinose at 79.4% and 67.9%, respectively. As the fermentation temperature continued to increase, the inhibition of enzyme activity related to yeast metabolism caused by higher temperatures affected yeast growth, which reduced the ability of yeast to metabolize and utilize sugar in soy molasses and led to an increase in the retention rates of the three sugars. It was worth noting that the optimal temperature for *W. anomalus* YT312 to bio-purify FOS in soy molasses was slightly higher than its optimal growth temperature in the YPD medium. It was speculated that the metabolic utilization of sucrose-, stachyose-, and raffinose-related enzymes in soy molasses was more favorable for expression or had higher enzyme activity at higher temperatures. Analogous to the findings of this investigation, prior research showed that the ideal temperature range for yeast growth and adaption for use in the bio-purification of soy molasses and the generation of FSO was normally 30 °C [35,50].

#### 3.2.3. Effect of Shaking Speed on Soy Molasses Purification by *W. anomalus* YT312

Yeasts are facultative anaerobic microorganisms that thrive under aerobic conditions. In the flask incubation method, shaking speed affected the oxygen content in the fermentation medium, thereby influencing yeast growth. Additionally, shaking speed generated shear force, which caused damage to yeast cells [49]. Higher shaking speeds were beneficial for increasing the oxygen content in the medium, but they also increased shear force, necessitating the optimization of the shaking speed. As shown in Figure 4c, after 12 h of static fermentation, there were no changes in the content of sucrose, raffinose, and stachyose in soy molasses, and only a small amount of manninotriose was detected. This was mainly because *W. anomalus* YT312 grew slowly and had a low cell density under static fermentation conditions, as confirmed by the growth curve results. With increasing shaking speed, the content of the three sugars decreased. The rate of sucrose reduction was higher than that of stachyose and raffinose. When the shaking speed was higher than 100 rpm, the sucrose in soy molasses was completely consumed by *W. anomalus* YT312, and the content of stachyose and raffinose was slightly decreased but still relatively high. Additionally, there was a significant increase in manninotriose content. Considering the retention rates of sucrose, stachyose, and raffinose, as well as the manninotriose content, the optimal shaking speed was 100 rpm, which is similar to the shaking speed used in the study by Liu et al. [35]. At this speed, the retention rate of sucrose was 0.0%, while the retention rates of stachyose and raffinose were as high as 79.3% and 90.2%, respectively. And the manninotriose content was 44.1 mg/mL.

#### 3.2.4. Effect of Dilution Ratio on the Soy Molasses Purification by *W. anomalus* YT312

Soy molasses is a byproduct of SPC production and contains a high concentration of sugar. While it provides sufficient nutrients for the growth of yeast, the high concentration of sugars creates a high osmotic pressure, which inhibits yeast growth to some extent. The results of sugar tolerance tests showed that although *W. anomalus* YT312 tolerated higher sugar concentrations, there was still a noticeable inhibitory effect at higher sugar concentrations. Therefore, the appropriate dilution of soy molasses was helpful for the biological purification and production of FSO by *W. anomalus* YT312. From Figure 4d, it was observed that at lower dilution ratios (four-fold and six-fold dilution), the high osmotic pressure affected yeast growth. At this point, the retention rates of sucrose and stachyose were higher, while the content of manninotriose was lower. Even though the pH of the diluted soy molasses was adjusted prior to sterilization, it was noteworthy that at these dilution ratios, the retention rate of raffinose was lower. This was caused by the acid hydrolysis of raffinose by some acidic components in soy molasses during high-temperature sterilization. The effect of this acid hydrolysis on stachyose was less evident. This phenomenon was not observed in the study by Zhang et al., which might be due to differences in soy molasses raw materials and sterilization processes [48]. As the dilution ratio increased, the osmotic pressure decreased, and the yeast growth improved gradually. Sucrose was completely consumed, while the content of raffinose initially increased and then decreased, transitioning from the effect of acid hydrolysis to yeast utilization. The content of stachyose showed a decreasing trend, and correspondingly, the content of manninotriose continued to increase. Through comprehensive analysis, it was concluded that the optimal dilution ratio for *W. anomalus* YT312 to biologically purify soy molasses was eight-fold. At this dilution ratio, the sucrose retention rate was 0.0%, the manninotriose content was 44.1 mg/mL, and the retention rates of stachyose and raffinose were 79.3% and 90.2%, respectively. This was consistent with the results of a yeast strain No. 7 and higher than the dilution ratio for a maltose yeast strain, which might be due to differences in yeast tolerance and the utilization of sugars [34,48].

#### 3.2.5. Effect of pH Value on the Soy Molasses Purification by *W. anomalus* YT312

The pH value of soy molasses not only affected the growth and reproduction of *W. anomalus* YT312 for FSO purification but also caused the acid hydrolysis of sugar in soy molasses. Therefore, it was crucial to adjust the pH of soy molasses appropriately. As shown in Figure 4e, with an increase in pH, the sucrose retention rate first rises, then falls, and then rises again. This was because, at lower pH values (such as pH 3), sucrose in soy molasses undergoes complete acid hydrolysis under high-temperature conditions. As the pH increased, the degree of acid hydrolysis decreased, leading to a higher retention rate. However, at higher pH values, the growth of *W. anomalus* YT312 was accelerated, resulting in a decrease in the sucrose retention rate. At pH 6.0, all sucrose in soy molasses was consumed. When the pH exceeded 6.0, yeast growth was inhibited, resulting in a decrease in the biological purification capacity and an increase in the sucrose retention rate. The retention rates of stachyose and raffinose showed a similar trend, increasing with an increase in pH. This was the result of pH having a dual effect on acid hydrolysis and yeast growth. The content of manninotriose showed a corresponding decrease. Yeast strain No. 7, *S. cerevisiae* 103, and *S. cerevisiae* 1607 all have an optimal pH of 5.0 for the biological purification of soy molasses to produce FSO, which was different from *W. anomalus* YT312. This difference might be due to variations in soy molasses raw materials and yeast strains [34,35,36].

#### 3.2.6. Effect of Fermentation Time on the Soy Molasses Purification by *W. anomalus* YT312

An appropriate fermentation time was essential for the purification of soy molasses by *W. anomalus* YT312 to prepare FSO. It was necessary to ensure the significant consumption of sucrose in soy molasses while maintaining high retention rates of stachyose and raffinose. As shown in Figure 4f, with the extension of fermentation time, *W. anomalus* YT312 transitioned from the lag phase to the logarithmic growth phase, with cells undergoing significant growth and reproduction. This led to the consumption of sucrose, stachyose, and raffinose in soy molasses, resulting in a decrease in their respective contents, while the content of manninotriose showed an increasing trend. After fermenting for 12 h, the sucrose retention rate reached 0, while the retention rates of stachyose and raffinose were 79.32% and 90.19%, respectively. The manninotriose content at this point was lower at 44.07 mg/mL. The best purification effect through fermentation was achieved at this time, which was similar to the time required for yeast strain No. 7 and *S. cerevisiae* 103 to purify soy molasses but shorter than that of maltose yeast and *S. cerevisiae* 1607. This was undoubtedly related to the composition of soy molasses and the characteristics of the yeast [34,35,36,48].

### 3.3. Optimization of Fermentation Conditions for Soy Molasses Purification by Orthogonal Experiment

The range values ω, τ, φ, and σ of the sucrose retention rate, raffinose retention rate, stachyose retention rate, and manninotriose content were calculated according to the above-mentioned formulas, and the results are shown in Table 4. The weights of sucrose, raffinose, stachyose, and manninotriose were calculated based on their respective concentration and weight formulas, with weights of 0.5, 0.1, 0.39, and 0.01, respectively. Based on the range value and weights, the comprehensive indicators K under each experimental condition were calculated and are shown in Table 4. According to the table, the best biological purification effect of *W. anomalus* YT312 on soy molasses was achieved under the experimental condition of a combination of A1B2C1D1E3F3 with an inoculum size of 0.125%, a fermentation temperature of 30 °C, six-fold dilution, a pH of 7, a shaking speed of 50 rpm, and a 14 h fermentation time. At these conditions, the sucrose, raffinose, and stachyose retention rates were 23.3%, 86.2%, and 92.0%, respectively, and the manninotriose content was 1.6 mg/mL. Variance analysis was performed using SPSS (Table 5), and the results showed that time had a significant impact on the biological purification of soy molasses by *W. anomalus* YT312, while other factors were not significant, with the influence order of F > E > D > A > C > B.

**Figure 5:**
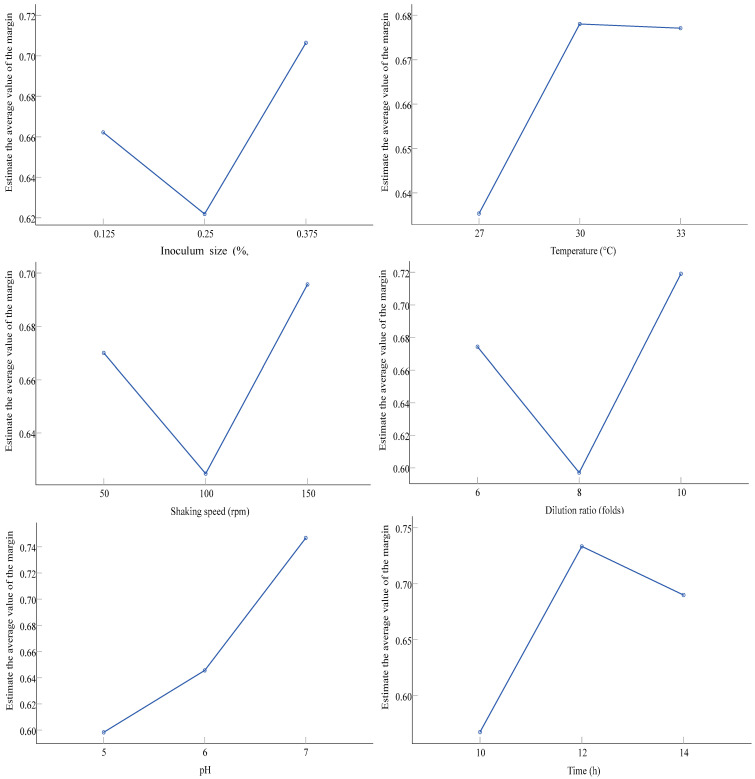
The contour plots of the comprehensive index for each factor of orthogonal experiment.

From the contour plots of the comprehensive index for each factor (Figure 5), it was evident that under the conditions of A3B2C3D3E3F2, *W. anomalus* YT312 exhibited a better bio-purification of soy molasses. The validation of this condition showed that under the A3B2C3D3E3F2 combination condition with an inoculum size of 0.375%, a fermentation temperature of 30 °C, a ten-fold dilution, a pH of 7, a shaking speed of 150 rpm, and a 12 h fermentation time, the retention rates of sucrose, raffinose, and stachyose in soy molasses were 0.0%, 96.1%, and 90.2%, respectively, with a manninotriose content of 2.9 mg/mL. Therefore, it was evident that under the A3B2C3D3E3F2 combination condition, the purification of soy molasses by *W. anomalus* YT312 for the preparation of FOS was ideal. The results were slightly inferior to those of yeast strain No. 7 in terms of raffinose and stachyose retention rates but more comprehensive in terms of sucrose removal [34]. Furthermore, the overall effect was better than that of *S. cerevisiae* 103, *S. cerevisiae* 1607, and maltose yeast [35,36,48].

## 4. Conclusions

In this study, strain YT312 was identified through morphological observation, physiological and biochemical tests, and molecular biology techniques. It was identified as *W. anomalus*, which exhibited a wide range of temperature and pH tolerances. It showed tolerances of 47.3% for sucrose, 41.2% for glucose, and 10% for NaCl. Through single-factor and orthogonal experiment optimization, the following conditions were determined to be optimal for soy molasses fermentation: an inoculum size of 0.375%, fermentation temperature of 30 °C, shaking speed of 150 rpm, dilution ratio of 10 times, pH of 7, and fermentation time of 12 h. Under these conditions, the retention rates of sucrose, raffinose, and stachyose in soy molasses were 0.0%, 96.1%, and 90.2%, respectively, and the content of manninotriose was 2.9 mg/mL. This is a yeast strain that is different from previously reported ones and can be used for the preparation of functional soy oligosaccharides from soy molasses. Yeast YT312 completely removes sucrose from soy molasses and has a high retention rate of FSO. This indicated a significant biological purification effect and the production of FSO through the fermentation of soy molasses by *W. anomalus* YT312. Future research may examine if it has an additional influence on the purification of other useful components, such as soy isoflavones in soy molasses, and whether it can convert sucrose into organic acids or important flavoring chemicals during the purification process.

## Figures and Tables

**Table 1:** Factors and levels of single-factor design and their optimization conditions.

Factor	Level
Inoculum size (%, *v*/*v*)	0.05, 0.125, 0.25, 0.375, 0.5, 1.0, 2.0, 4.0, 6.0, 8.0, and 10.0
Temperature (°C)	15, 18, 21, 24, 27, 30, 33, 36, and 39
Shaking speed (rpm)	0, 50, 100, 150, 200, 250, and 300
Dilution ratio (folds)	2, 4, 6, 8, 10, 12, 14, and 16
pH	3, 4, 5, 6, 7, 8, 9, and 10
Time (h)	2, 4, 6, 8, 10, 12, 14, and 16

**Table 2:** Design of orthogonal experiment.

Experiment Group	Inoculum Size (A) (%, *v*/*v*)	Temperature (B) (°C)	Shaking Speed (C) (rpm)	Dilution Ratio (D) (Folds)	pH (E)	Time (F) (h)	Blank
1	0.125 (1)	27 (1)	50 (1)	6 (1)	5 (1)	10 (1)	1
2	0.125	30 (2)	100 (2)	8 (2)	6 (2)	12 (2)	2
3	0.125	33 (3)	150 (3)	10 (3)	7 (3)	14 (3)	3
4	0.25 (2)	27	50	8	6	14	3
5	0.25	30	100	10	7	10	1
6	0.25	33	150	6	5	12	2
7	0.375 (3)	27	100	6	7	12	3
8	0.375	30	150	8	5	14	1
9	0.375	33	50	10	6	10	2
10	0.125	27	150	10	6	12	1
11	0.125	30	50	6	7	14	2
12	0.125	33	100	8	5	10	3
13	0.25	27	100	10	5	14	2
14	0.25	30	150	6	6	10	3
15	0.25	33	50	8	7	12	1
16	0.375	27	150	8	7	10	2
17	0.375	30	50	10	5	12	3
18	0.375	33	100	6	6	14	1

**Table 3:** Results of physiological and biochemical characteristics of *W. anomalus* YT312.

Sugar Fermentation Test		Carbon Source Assimilation Test		Nitrogen Source Assimilation Test		Others Test	
Sugar	Characteristics	Carbon Source	Characteristics	Nitrogen Source	Characteristics	Test	Result
Saccharose	Acid and gas production; growth	Soluble starch	+	Urea	+	Hydrogen sulfide test	−
Maltose	Not all produce acid; gas production; growth	Ethyl alcohol	+	Potassium nitrate	+	Indole test	+
Xylose	No acid and gas; growth	Mannitol	+	Potassium nitrite	+	Urea test	−
Lactose	No acid and gas; growth	Citric acid	+	L-Phenylalanine	+	Methyl red test	+
Galactose	Not all produce acid; gas production; growth	Rhamnose	+	Ammonium sulfate	+	Voges–Proskauer test	−
Arabinose	No acid and gas; growth	Trehalose	+			Gelatin liquefication test	−
Sorbinose	No acid and gas; growth	Formic acid	−			Citrate test	+
Glucose	Not all produce acid; gas production; growth	Glycerol	+			Starch hydrolysis test	−
		Fructose	+			Litmus milk test	+

Note: “+”, positive response; “−”, negative response.

**Table 4:** Optimization of soy molasses fermentation conditions for FSO production by orthogonal experiment.

Experiment Group	Sucrose		Raffinose		Stachyose		Manninotriose		K
	Retention Rate (%)	Range ω	Retention Rate (%)	Range τ	Retention Rate (%)	Range φ	Content (mg/mL)	Range σ	
1	85.3	0.07	85.0	0.86	91.0	0.91	2.7	0.99	0.49
2	63.7	0.30	83.4	0.83	95.0	0.97	2.2	0.99	0.62
3	0.2	1.00	38.8	0.34	80.1	0.75	54.6	0.32	0.83
4	45.8	0.50	80.9	0.80	71.0	0.61	37.1	0.55	0.57
5	45.6	0.50	66.8	0.65	96.2	0.99	2.7	0.99	0.71
6	18.7	0.80	33.8	0.26	84.9	0.82	3.2	0.98	0.76
7	23.5	0.74	32.6	0.26	89.2	0.89	1.6	1.00	0.75
8	0.0	1.00	11.9	0.02	52.2	0.33	29.9	0.64	0.64
9	46.5	0.49	33.4	0.26	95.1	0.97	2.7	0.99	0.66
10	30.8	0.66	75.8	0.73	96.8	1.00	2.7	0.99	0.80
11	23.3	0.75	86.2	0.87	92.0	0.93	1.6	1.00	0.84
12	91.5	0.00	40.1	0.34	90.6	0.91	2.2	0.99	0.40
13	0.0	1.00	35.0	0.28	29.8	0.00	79.8	0.00	0.53
14	71.4	0.22	60.8	0.57	81.8	0.78	8.3	0.91	0.48
15	41.2	0.55	96.6	0.99	81.4	0.77	11.6	0.87	0.68
16	29.7	0.68	54.3	0.51	75.9	0.69	12.1	0.87	0.67
17	4.4	0.95	49.5	0.45	73.8	0.66	28.4	0.66	0.78
18	10.3	0.89	73.0	0.72	65.9	0.54	11.5	0.87	0.74

**Table 5:** Analysis of variance of orthogonal experiment.

Dependent Variable VAR00001	Tests of Between-Subject Effects				
	III Sum of Squares	Degree of Freedom	Mean Square	F	Significance
Corrected model	0.248	12	0.021	3.255	0.101
Intercept	7.924	1	7.924	1249.081	0.000
Inoculum size (A)	0.021	2	0.011	1.691	0.275
Temperature (B)	0.007	2	0.004	0.563	0.602
Shaking speed (C)	0.016	2	0.008	1.222	0.370
Dilution ratio (D)	0.046	2	0.023	3.607	0.107
pH (E)	0.069	2	0.035	5.443	0.056
Time (F)	0.089	2	0.044	7.008	0.035 *
Error	0.032	5	0.006		
Total	8.204	18			
Corrected total	0.280	17			
R^2^ = 0.887 (Adjusted R^2^ = 0.614)					

Note: “*”, significant difference (*p* < 0.05).

## Data Availability

The data presented in this study are available on request from the corresponding author. The data are not publicly available due to privacy restrictions.
